# Once small always small? To what extent morphometric characteristics and post-weaning starter regime affect pig lifetime growth performance

**DOI:** 10.1186/s40813-018-0098-1

**Published:** 2018-07-23

**Authors:** A. M. S. Huting, P. Sakkas, I. Wellock, K. Almond, I. Kyriazakis

**Affiliations:** 1 Agriculture, School of Natural and Environmental Sciences, Newcastle University, Newcastle Upon Tyne, NE1 7RU UK; 2 Primary Diets, ABAgri, Melmerby, Ripon, North Yorkshire HG4 5HP UK

**Keywords:** Birth weight, Body mass index, Growth, Pigs, Ponderal index, Weaning weight

## Abstract

**Background:**

The aim of this study was to determine the effect of piglet morphometric characteristics and starter regime on postnatal growth. Some piglets born light are able to grow faster than others, and identifying which piglets are more at risk to remain light and at which stages of growth is essential. A nutrient enriched starter regime may allow lightweight pigs to improve their post-weaning growth. A total 1487 newly born piglets from 137 litters originating from 8 consecutive farrowing batches were followed from birth (BiW) to weaning (WW, d28) and finishing (d99). At birth morphometric measurements were taken, including body mass index (BMI), ponderal index (PI) and BiW:cranial circumferences (BiW:CC). At weaning pigs were randomly allocated to one of two experimental regimes: either a nutrient enriched regime with a 20% higher essential amino acids (EAA): energy ratio (HIGH) or a standard regime (CTRL). Piglets were retrospectively allocated to 4 different weight classes (C) using percentiles at birth, weaning and finishing, with C1 representing the lightest and C4 the heaviest class. A series of novel statistical models were used to determine which factors were able to predict performance.

**Results:**

For BiW C1 piglets, BMI (*P* = 0.003) and BiW relative to birth litter (*P* = 0.026) were positively associated with pre-weaning performance, whereas BiW:CC (*P* = 0.011) and WW (*P* = 0.001) were positively associated with post-weaning growth. Post-weaning the best predictors of piglets weaned light (WW C1) were PI (*P* = 0.037), BiW:CC (*P* < 0.001) and WW (*P* < 0.001). Starter regime did not influence (*P* > 0.05) post-weaning performance.

**Conclusion:**

Our results show that not all light pigs are the same and that their performance is under the influence of body shape rather than BiW. Therefore, pig producers should discriminate between light pigs based on birth characteristics to improve the effectiveness of intervention strategies at the different stages of growth. Irrespective of weight class piglets did not benefit from the EAA enriched regime applied.

**Electronic supplementary material:**

The online version of this article (10.1186/s40813-018-0098-1) contains supplementary material, which is available to authorized users.

## Background

Slow growing pigs are more at risk to be delayed in all-in-all-out systems, resulting in remixing, increasing the potential for disease transmission, but most importantly contributing to considerable production losses (e.g. costs of feed, labour and penalties at slaughter) [1]. However, it has been suggested that some pigs born light may have the potential to compensate during suckling [2] and subsequent growth stages [3–5]. It is therefore important to identify which pigs are most likely to remain light throughout the production cycle and may require attention. Birth- [1, 2] and weaning-weight [3–6] have been identified as predictors for post-weaning growth. Morphometric characteristics at birth predict survivability [7, 8] and may be utilised to identify piglets that remain stunted throughout life [9], or potentially benefit from intervention strategies. However, the evidence about the effect of body shape at birth on subsequent performance is scarce and lacks a life time performance approach.

One strategy that has been shown to be effective in improving the performance of lightweight pigs are high specification starter regimes [10, 11]; pigs weaned light have a poor start, but under the influence of an improved nutritional regime may be able to improve their performance. However, the regimes studied previously [10, 11] have altered the ingredient composition considerably, making it impossible to identify which specific nutrients resource(s) would be most beneficial. Slow growing pigs are suggested to have a lower feed intake and lower serum concentrations of essential amino acids (EAA) [5] compared to their fast growing siblings. The low feed intake of lightweight pigs [12, 13] and possibly higher protein turnover in relation to their size [14], suggest that lightweight pigs may exhibit improved performance when fed nutrient enriched diets that are high in EAA [15]. The objectives of this study were: 1) to assess the influence of morphometric characteristics at birth on performance to finisher stage and whether these can differentiate between pigs that are able to exhibit an improved performance pre- and post-weaning; and 2) whether a nutrient enriched starter regime could contribute to an improved post-weaning performance of piglets weaned light.

## Methods

The experiment was conducted at Cockle Park Farm (Newcastle University, Morpeth, Northumberland, United Kingdom). All animals were maintained in accordance to the recommendations for the welfare of livestock following UK legislations (Defra and Red Tractor UK farm assurance scheme) and the experiment was approved by the Animal Welfare and Ethical Review Body (AWERB project ID no. 419) of Newcastle University. All newly born piglets (*n* = 1487) of the 137 sows that farrowed during 8 consecutive farrowing batches were followed to finisher stage (~ 14 weeks of age and 45 kg BW).

### Pre-weaning management

Following a 3-week cycle, sows of different parity were moved on Monday to the farrowing unit; those that had not farrowed by Thursday, were induced (23.9% of the sows) with a Prostaglandin analogue (Planate; Intervet UK, Walton, United Kingdom). All sows were Large White x Landrace, inseminated with Hylean boar semen (Hermitage Seaborough, Ltd., Devon United Kingdom). They were fed a home-milled meal twice a day (08:00 and 15:00 h) and water was available ad libitum throughout lactation. The temperature in the farrowing unit was maintained at 21 °C (20.7 °C, range 18.2 to 26.9 °C).

AHDB Pork guidelines for cross fostering [16] were followed, to help piglets born light reduce competition and fit piglet mouths to the teat size of the sow. Cross fostering was applied within the first 3 days post-partum to improve litter uniformity and to equalize litter size matching the number of piglets with the number of functional teats and milking ability of the sow (litter size range 10 to 15). During the first two days of life piglets were locked into the creep area (once a day; 08:00 h), whilst the sow was eating, to minimize crushing. An infrared heat lamp was located in the covered creep area and wood shavings were provided as bedding. Piglets had unlimited access to a water nipple drinker. Within the first 12 h after birth piglets had their teeth clipped. At ~ 3 days of age, piglets were tail docked and received an intramuscular iron injection. All piglets had access to creep feed from 10 days of age which was fed in small quantities (a handful) on the floor of the covered creep area. The creep was an equal mixture (50:50) of diet 1 of the standard (**CTRL**) and nutrient enriched (**HIGH**) starter regimes (see section ‘Post-weaning starter regime’).

### Post-weaning management

Piglets were weaned at approximately 28 days of age (d 27.7, SD = 1.07) and were vaccinated for *M. hyopneumoniae* (M + PAC; Intervet UK, Walton, United Kingdom) and porcine circovirus type 2 (Inglevac Mycoflex; Boehringer Ingelheim, Ingelheim, Germany). They were randomly mixed to form groups of approximately 20 similar sized pigs/pen (range 9 to 24 pigs/pen), whilst balancing for gender, and moved to a fully slatted nursery accommodation. The number of pigs per pen was dependent on the number of pigs available per batch, ensuring a similar stocking density between pens and batches consistent with UK legislations. The nursery accommodation consisted of 6 separate environmental rooms, with approximately 3 rooms per batch. Pen size, where appropriate, was adjusted creating a minimum of 4 to a maximum of 8 pens per room. All pigs had ad libitum access to water via nipple drinkers. The initial room temperature in the nursery accommodation was set at 26 °C (24.6 °C, range 20.7 to 27.2 °C) and reduced by approximately 0.2 °C each day to a minimum of 22 °C (23.4 °C, range 21.5 to 26.7 °C).

When moved to the on-site grower accommodation (d 61, SD = 1.17), pigs were fed the same home-milled meal and remained in the same post-weaning group. Groups of < 12 pigs were mixed to create groups of ~ 20 pigs/ pen. At approximately 13–14 weeks of age (d 96.9, SD = 6.63) pigs were moved again to a fully slatted finisher building and were fed a commercial ‘finisher’ pelleted diet.

### Experimental procedures

#### Pre-weaning procedures

Piglets were weighed to the nearest 1 g within 12 h post-partum (**BiW**, kg), and individually identified (ID) by ear tagging (Dentag, Toptags, Kelso, UK). Morphometric measurements were taken from each individual piglet, including crown to rump length (**CRL**, cm), snout to ears length (head length **HL**, cm), abdominal circumferences (**AC**, cm) and cranial circumferences (**CC**, cm). Abdominal circumferences, was taken at the anterior side of the umbilicus cord. Crown rump length (m) was used to calculate the ponderal index (**PI**; BiW, kg/CRL, m^3^) and body mass index (**BMI**; BiW, kg/CRL, m^2^) [9]. Additional variables were created as an indicator of head size in relation to body weight: 1) birth weight: cranial circumferences (**BiW:CC**, kg/cm) [9] and 2) snout to ears length: birth weight (**HL:BiW**, cm/kg) [17].

At the point of tail docking litter composition (foster litter), including sow and piglet ID, was recorded. The general health of piglets was examined on a daily basis and deaths, including cause of death where possible, were recorded. Piglet ear tags were replaced by larger ear tags (Suretag flag, Dalton tags, Newark Nottinghamshire, UK) and piglets were individually weighed at weaning (**WW**, d 27.7, SD = 1.07).

#### Post-weaning starter regime

At weaning, piglets were randomly allocated to either a nutrient enriched (HIGH) or a control (CTRL) 3-stage starter regime (Primary Diets, ABAgri, Ripon, North Yorkshire, UK). Diet 1 and 2 of the HIGH regime (Table 1) were supplemented with additional synthetic EAA L-lysine, DL-methionine, L-threonine, L-tryptophan and L-valine, in order to achieve 20% higher EAA: energy ratio when compared to the CTRL diet, while maintaining the same NE and ensuring the appropriate ratios to lysine were maintained; the CTRL regime met NRC recommendations [18]. By 7 weeks of age (d 48.0, SD = 0.887) all pigs had finished the first 2 starter diets. Diet 3 of both the HIGH and CTRL regimes were identical.

**Table 1:** Ingredient composition on an as-fed basis and chemical analysis of the post weaner feeds used. Pigs were randomly allocated to either a nutrient enriched (HIGH) or a standard (CTRL) starter regime. Diet 1 was fed until 2 kg was consumed, and diet 2 until 3 kg were consumed per pig. Diet 3 was fed *ad libitum*^1^

*Diet*	1		2		3
*Regime*	CTRL	HIGH	CTRL	HIGH	
Ingredient g/kg					
Micronized barley	75.0	75.0	75.0	75.0	100.0
Wheat	105.0	93.8	365.1	353.7	529.7
Micronized wheat	150.0	150.0	50.0	50.0	–
Micronized oats	100.0	100.0	50.0	50.0	–
Fishmeal	75.0	75.0	50.0	50.0	25.0
Soya bean meal	160.0	160.0	220.0	220.0	260.0
Pig weaner vitamin/	5.0	5.0	5.0	5.0	5.0
Trace element supplement^2^					
Dried skim milk powder	75.0	75.0	30.0	30.0	–
Whey	225.7	225.7	118.1	118.1	34.7
L-Lysine HCL	1.80	5.70	3.10	6.80	3.70
DL-Methionine	1.70	2.90	2.00	3.20	2.10
L-Threonine	0.50	2.40	0.90	2.70	1.10
L-Tryptophan	0.20	0.80	0.20	0.80	0.10
L-Valine	0.00	2.30	0.40	2.50	0.40
Vitamin E	0.04	0.40	0.20	0.20	0.20
Benzoic acid	5.00	5.00	5.00	5.00	5.00
Limestone flour	–	–	1.30	1.30	–
Dicalcium phosphate	1.80	1.80	7.30	7.30	15.1
Salt	–	–	1.40	1.60	3.60
Binder (LignoBond DD)^3^	–	–	–	–	4.20
Sodium bicarbonate	0.10	1.90	5.00	6.80	–
Soya oil	17.3	17.2	10.0	10.0	10.1
Analysed composition, % as fed					
CP	21.1	22.1	21.9	22.8	21.5
Crude fiber	1.85	1.95	2.20	2.10	2.45
Moisture	10.4	9.70	10.0	10.1	11.0
Ash	4.75	5.15	6.10	6.40	5.60
Calculated composition, % as fed or as specified					
DE, MJ/kg	15.3	15.3	14.7	14.8	14.4
NE, MJ/kg	10.5	10.5	10.2	10.2	10.1
Calcium	0.77	0.77	0.77	0.77	0.75
Phosphorus	0.66	0.66	0.64	0.64	0.67
Lactose	20.0	20.0	10.0	10.0	2.50
Lysine^4^	1.40	1.70	1.35	1.64	1.25
Methionine	0.56	0.68	0.54	0.66	0.50
Methionine + Cysteine	0.84	0.96	0.83	0.94	0.79
Threonine	0.84	1.02	0.81	0.98	0.75
Tryptophan	0.27	0.32	0.26	0.31	0.24
Arginine	1.15	1.15	1.20	1.20	1.20
Histidine	0.50	0.50	0.49	0.48	0.47
Isoleucine	0.87	0.87	0.82	0.82	0.76
Leucine	1.56	1.55	1.44	1.43	1.32
Valine	0.98	1.19	0.95	1.15	0.88
Phenylalanine + Tyrosine	1.57	1.56	1.54	1.53	1.48

^1^Diets were supplied by Primary Diets, ABAgri, Ripon, North Yorkshire, United Kingdom

^2^It provided per kilogram of complete diet 11,500 IU of vitamin A, 2000 IU of vitamin D_3_, 100 IU of vitamin E, 4 mg of Vitamin K, 27.5 μg of vitamin B_12_, 15 mg of pantothenic acid, 25 mg of nicotinic acid, 150 μg of biotin, 1.0 mg of folic acid, 160 mg of Cu (CuSO_4_), 1.0 mg of iodine (Ca (IO_3_)_2_), 150 mg of Fe (FeSO_4_), 40 mg of Mn (MnO), 0.25 mg of Se (bone morphogenetic protein), and 110 mg Zn (ZnSO_4_)

^3^Borregaard LignoTech, Sarpsborg, Norway

^4^All amino acids are expressed on a standardized ileal digestible (SID) basis

For either regime, diet 1 was fed until 2 kg of feed were consumed and diet 2 until 3 kg of feed were consumed per pig. Diet 3 was fed ad libitum up to 9 weeks of age when pigs were moved to the grower accommodation. A total of 70 nursery pens of animals were part of the experiment; 36 pens (*n* = 679) were fed the CTRL and 34 pens (*n* = 683) were fed the HIGH starter regime.

#### Post-weaning procedures

Until movement to the grower facility (d 61.5, SD = 1.17), pigs were weighed once a week. At the same time the amount of feed offered and refused was recorded to estimate weekly feed intake. Pigs that lost weight during the first week post-weaning were weighed individually and daily during two successive days; those that kept losing BW were removed from the experiment (see ‘Statistical Analysis’ section as to how this was addressed in the final models). Two hundred and six pigs (15.5%) were sold as growers (d 74.8, SD = 1.93). The remainder of the pigs (*n* = 1121) were individually weighed at finisher stage (d 98.8, SD = 0.937).

### Statistical analysis

All statistical models were performed with SAS version 9.4 (SAS inst. Inc. Cary, NC) using mixed models (PROC MIXED) unless stated otherwise. The residual variance of the data was tested for normality using the UNIVARIATE procedure. Several covariance structures were tested, but variance components resulted in the lowest Akaike Information Criterion (AIC) with an AIC difference of > 4 considered substantial [19]. Data were expressed as least square means (LSM), with approximate standard errors of the differences of means (SED) unless stated otherwise. Statistical significance was assessed at the 5% level and tendencies were set at 10%.

#### Performance per pen

The effect of starter regime on post-weaning performance and coefficient of variation (CV) within a pen was assessed using PROC MIXED. The experimental unit was pen average blocked by room nested within farrowing batch; a weight statement was used to account for differences in the number of pigs per pen. Gender was added to the preliminary model as covariate.

#### Absolute performance per body weight class

Pigs were retrospectively assigned to body weight (BW) classes based on 25% percentiles [3] creating 4 groups at birth (**BiW**), weaning (**WW**) and finisher (**FW**). Class 1 represented the lightest (bottom 25%) and 4 the heaviest (top 25%) pigs. Body weight classes created included all pigs that were alive or remained on site at the start of a specific stage of production (e.g. birth, weaning and finisher). Classes were created both within batch and over the entire period, however classes that were created within batch resulted in the best model fit. The experimental unit for all mixed models was piglet, blocked by farrowing batch. To account for any litter or pen effects piglets were blocked by: (1) sow (birth or foster sow) nested within farrowing batch for evaluating pre-weaning performance, and (2) pen x room nested within farrowing batch for evaluating post-weaning performance. In the post-weaning mixed model main effects of interest were BW class, starter regime and their interaction. As classes were created retrospectively and piglets were allocated to the different starter regimes on the basis of their WW and not BiW, WW was inserted in the model for BiW class to account for WW differences at the start of the treatment. In the preliminary models farrowing batch, gender, total number of pigs born, birth litter/ parity, foster litter/ parity, pre-weaning litter size, age and post-weaning group size were inserted as covariates where appropriate. As a result of mortality and pig removals due to weight loss or sickness, pre-weaning litter and post-weaning group size were corrected using the following formula over a given period:

Litter/Group size = [(total time (h) piglets reside in the foster litter/ pen)/ 24 h]/ total period in d

An additional variable ‘pen variation’ was created (pen variation = average BW class within pen) based on BiW or WW class and was added to all post-weaning analysis. Additional file 1: Table S1 specifies the final model descriptions after removal of nonsignificant covariates used for the different objectives.

A chi-square test was carried out to test whether the reason for removal and pre- and post-weaning mortality was different among pigs of different BW classes and whether this was affected by starter regime and/or gender. In addition, a chi-square was used to test whether the number of pigs that decreased, remained or increased at least one BW class at weaner or finisher [3, 20] was different among the different BW classes.

#### Cluster analysis

A principal component analysis (PCA) was performed using PROC FACTOR to determine whether there is a distinct group within BiW and WW class 1 piglets able for compensatory growth during respectively the pre- and post-weaning period. An additional variable for birth weight in relation to birth litter average were calculated using the following formula [4, 8]:

Relative birth weight (**Relative BiW**): [Birth weight piglet/ mean birth weight birth litter]

For BiW class 1 piglets 10 variables were considered, including birth weight, relative BiW and the various morphometric characteristics (AC, CC, CRL, BMI, PI, HL, BiW: CC and HL: BiW). The above variables plus pre-weaning ADG and WW were considered for WW class 1 piglets. Principal components with an Eigenvalue greater than 1 were retained in the model and were used in the cluster analysis (PROC CLUSTER) using the Ward method to minimise within-cluster variance. The number of clusters were determined on the basis of fit statistics (e.g. Cubic Clustering Criteria, Pseudo *F* and *t*^*2*^ statistics; [21]) and the dendrogram. The effect of the clusters on the different variables that were considered in the PCA and its effect on pre- and post-weaning performance were analysed using mixed models adjusting the degrees of freedom to unequal variance with denominator degrees-of-freedom (DDF) Satterthwaite and studentized maximum modulus (SMM) enabling multiple comparison.

#### Probability for compensatory growth

Two different logistic regressions (PROC LOGISTIC) were conducted to identify whether piglets from different BW classes differ in their ability to change class in later life, and whether this was under the influence of starter regime and the various morphometric characteristics. The first logistic regression tested whether piglets of BiW class 1 to 4 had a different probability to end up in WW class 1 to 4. A similar model was conducted between BiW – FW and WW - FW. The effects of interest was BW class (BiW or WW class), starter regime and their interaction. In the second logistic regression, pre-weaning ADG and various morphometric characteristics were added to determine whether pig ability to change BW class decreased or increased (log odds ± SE) with one unit increase in the predictor variable. For both regressions, the response variable of interest (e.g. WW and FW class) had more than two levels and was therefore formatted to enable the estimation of piglet probability to end up in one of the intermediate BW classes (class 2 or 3), with zero representing everything other than the BW class of interest. The reference value was set to the final BW class of interest using the DESCENDING option to ensure the likelihood to end up in the ‘highest’ BW class was tested.

#### Multivariate analysis

All potential predictor variables were fitted in a univariate mixed model to test their effect on pre- and post-weaning performance. Only predictor variables that were significant (*P* < 0.05) in the univariate model were taken forward in the multivariate analysis. Multivariate models were built following a forward and backward stepwise procedure only leaving factors in that had a probability below 0.05 and using the AIC criteria to determine which model fitted best. Different models were built for variables that were highly correlated (*r* > 0.70) to ensure the variance inflation factor (PROC REG) remained low (< 2).

## Results

An overview of pre-and post-weaning farm characteristics can be found in Additional file 2: Table S2. Gender significantly affected birth (*P* = 0.006) and weaning weight (*P* = 0.043), with males being weaned heavier (respectively 1.47 kg, SD = 0.332 and 7.26 kg, 2.20) than females (respectively 1.46 kg, SD = 0.350 and 7.09 kg, SD = 2.08). Certain morphometric characteristics, including AC, CC, PI, and BMI, were also significantly (at most *P* < 0.05) lower for females than male piglets. However, the ratio of head size to BW (i.e. BiW:CC) tended (*P* = 0.094) to be higher in females than in male piglets. Weak positive correlations were found between BiW and WW (*r* = 0.497, *P* < 0.001) and BiW and pre-weaning ADG (*r* = 0.326, *P* < 0.001), whereas a high correlation was found between WW and pre-weaning ADG (*r* = 0.970, *P* < 0.001).

The average BW for the different FW classes (d 98.8, SD = 0.938) was 36.5 kg (SD = 3.14), 43.1 kg (SD = 3.07), 47.4 kg (SD = 3.08), and 53.3 kg (SD = 3.23) for classes 1–4 respectively.

### Performance per pen

Gender was equally distributed across treatments (*P* > 0.05); WW and pen CV (d 28) did not differ between starting regimes. Post-weaning performance (d 28 to 61) was not affected by starter regime; BW and pen CV at various stages of production along with feed intake and gain to feed ratio were not significantly different (*P* > 0.05) between pigs allocated to the HIGH or CTRL regime.

### Absolute performance per birth weight class

Table 2 shows the total number of piglets per BiW class at the different stages of production. The highest pre-weaning mortality rate (21.1%) was observed for piglets born light (class 1), compared to piglets of BiW class 2–4 (*P* < 0.001). Most (46.8%) of these BiW class 1 piglets were non-viable at birth or died of starvation, of which 67.6% were male and 32.4% female pigs (*P* = 0.003). The number of piglets per starter regime was unbalanced (*P* < 0.001) for BiW class 2 and 4 piglets. Significantly (*P* < 0.001) more piglets of BiW class 2 were allocated to the CTRL versus HIGH regime. The opposite was the case for piglets of BiW class 4 (*P* < 0.001). Within pen variation was not significantly different across treatments (*P* < 0.05). Total post-weaning removal (including weight loss, sickness, and mortality) was significantly affected by BiW class. Significantly (*P* = 0.028) more piglets of BiW class 1 (3.7, 72.7% males and 27.3% females, *P* = 0.033) were removed than piglets born heavy (class 4, 1.1%). Also, piglets of BiW class 2 tended (*P* = 0.065) to be removed in higher quantities compared to piglets of BiW class 4 (3.1% vs. 1.1%). Total post-weaning removal was affected by starter regime (*P* = 0.040), with the highest removal observed for piglets fed the HIGH regime (*n* = 19, 2.9%) compared to those fed the CTRL regime (*n* = 8, 1.2%).

**Table 2:** Total number of pigs pre- and post-weaning per birth weight class. Within batch birth weight classes were created retrospectively using percentiles (25%) resulting in 4 different groups. Class 1 represents the lightest piglets and class 4 the heaviest. At weaning pens were randomly allocated to one of the starter regimes: control (CTRL) vs. nutrient enriched starter regime (HIGH)

*Birth weight class*	1		2		3		4		Total		Significance^1^
*Starter regime*	CTRL	HIGH	CTRL	HIGH	CTRL	HIGH	CTRL	HIGH	CTRL	HIGH	
Number of pigs^2^											
Day 0	374		371		372		370		1487		1.000
Day 28	148	147	202^a^	150^b^	192	169	137^b^	217^a^	679	683	< 0.001
Day 48	146	139	199^a^	143^b^	190	167	137^b^	215^a^	672	664	< 0.001
Day 61	146	138	198^a^	143^b^	190	167	137^b^	215^a^	671	663	< 0.001
Day 97	142	133	189^a^	128^b^	176^a^	146^b^	115^b^	191^a^	564	557	< 0.001
Day 75	15^a^	5^b^	19	6	12	10	12	20	58	41	0.164
Day 99^3^	127	128	170^a^	122^b^	164^a^	136^b^	103^b^	171^a^	515	498	< 0.001

^a,b^Absolute values within birth weight class with different superscripts differ significantly (*P* < 0.05) between starter regimes

^1^A chi square test was used to test the overall difference between the different birth weight classes x starter regime (entire row, excluding total)

^2^Pigs were followed from birth (d 0), weaning (d 27.7, SD = 1.07), grower (d 61.5, SD = 1.17), to finisher (d 96.9, SD = 6.63). Two hundred and six pigs were sold as growers (32.6 kg, SD = 2.86) at an age of d 75 (d 74.8, SD = 1.94) of which 99 were weighed and the rest (*n* = 107) no additional weights were taken

^3^Only those pigs that reached finisher age on site (d 98.8, SD = 0.938)

Table 3a shows the effect of BiW class and starter regime on subsequent performance. Birth weight class significantly (*P* < 0.001) affected piglet BW throughout the productive period; pigs in class 1 remained lighter throughout the different stages of production, weighing > 3 kg lighter (40.9 kg, SD = 10.7) at d 97 than piglets born heavier (44.0 kg, SD = 10.5, 44.8 kg, SD = 10.4, and 45.6 kg, SD = 10.7 for respectively class 2, 3, and 4). Although starter regime did not affect BW at d 48 (*P* > 0.05), it tended to influence piglet BW at d 61 (*P* = 0.059). Piglets that were allocated to the CTRL regime weighed 1.00 kg heavier at the end of the nursery (23.1 kg, SD = 8.85) than piglets fed the HIGH regime (22.1 kg, SD = 8.69). However, at finisher (d 97) the effect of starter regime was absent (*P* > 0.05). Apart from WW (*P* < 0.001), there was no significant interaction between BiW class and starter regime at later stages of production (*P* > 0.05); the significant interaction at weaning was a result of diet and pig allocation: following normal farm practices piglets were grouped together on the basis of WW and not BiW.

**Table 3:** Effect of birth weight class (**A**), weaning weight (**B**) class and starter regime on subsequent performance. Per batch birth weight (BiW, d 0) and weaning weight (WW, d 28) class were determined retrospectively by grouping piglets into 4 different classes (25%) using percentiles at birth and weaning. Class 1 represents the lightest piglets and class 4 the heaviest. At weaning pens were randomly allocated to one of the starter regimes (Diet): control (CTRL) vs. nutrient enriched starter regime (HIGH). Data are expressed as LSM ± SED

A	Birth weight class	1		2		3		4		SED	Significance		
	Starter regime	CTRL	HIGH	CTRL	HIGH	CTRL	HIGH	CTRL	HIGH		BiW class	Diet	BiW class *Diet
	Body weight^1^, kg												
	d 0	1.02		1.37		1.58		1.87		0.001	< 0.001	–	–
	d 28	6.67^a^	6.01^b^	7.05	6.96	7.40	7.59	7.64^b^	8.09^a^	0.041	< 0.001	0.729	< 0.001
	d 48	14.1	13.6	14.6	14.5	14.9	15.0	15.4	15.3	0.039	< 0.001	0.312	0.139
	d 61	22.1	21.0	22.8	22.4	23.1	22.8	23.8	23.3	0.077	< 0.001	0.059	0.386
	d 97	42.5	40.6	44.6	43.7	44.6	44.7	44.8	45.1	0.197	< 0.001	0.514	0.369
	Average daily gain, g/d												
	d 0–28	186		202		213		218		0.413	< 0.001	–	–
	d 28–48	343	313	365	360	377	382	405	399	1.95	< 0.001	0.312	0.111
	d 48–61	586	547	611	584	611	582	622	593	3.00	< 0.001	0.036	0.993
	d 28–61	441	406	463	449	471	462	492	476	2.06	< 0.001	0.075	0.298
	d 61–97	580	552	615	590	606	609	606	620	3.04	< 0.001	0.520	0.302
B	Weaning weight class	1		2		3		4		SED	Significance		
	Starter regime	CTRL	HIGH	CTRL	HIGH	CTRL	HIGH	CTRL	HIGH		WW class	Diet	WW class *Diet
	Body weight^1^, kg												
	d 28	5.42	5.30	6.65	6.66	7.60	7.68	9.11	9.17	0.012	< 0.001	0.920	0.194
	d 48	12.6	12.4	14.3	14.3	15.2	15.0	16.7	16.9	0.046	< 0.001	0.825	0.747
	d 61	20.1	19.2	22.4	22.0	23.5	23.2	25.7	25.4	0.075	< 0.001	0.212	0.765
	d 97	40.1	39.6	43.9	43.3	45.8	44.0	47.3	47.4	0.204	< 0.001	0.436	0.462
	Average daily gain, g/d												
	d 28–48	339	331	379	380	378	372	387	392	2.09	< 0.001	0.825	0.776
	d 48–61	538	514	597	579	604	617	649	640	3.00	< 0.001	0.198	0.303
	d 28–61	423	399	467	454	473	465	497	491	2.18	< 0.001	0.219	0.737
	d 61–97	576	549	608	601	625	590	614	629	3.60	< 0.001	0.332	0.194

^a,b^Within BW class numbers with different superscripts differ significantly (*P* < 0.05)

^1^Pigs were weighed within 12 h post-partum (d 0), at weaning (d 27.7, SD = 1.07), 3 weeks post weaning (d 48.0, SD = 0.887), grower (d 61.5, SD = 1.17), and finisher (d 96.9, SD = 6.63)

### Absolute performance per weaning weight class

A total of 1362 piglets were weaned of which *n* = 355 were considered lightweight (WW class 1), *n* = 342 pigs belonged to WW class 2, *n* = 329 to WW class 3, and *n* = 336 to WW class 4 (*P* > 0.05). The number of pigs per starter regime within WW class was unbalanced (*P* < 0.001). Significantly (*P* = 0.001) more pigs of WW class 1 were allocated to the HIGH (*n* = 155) versus CTRL regime (*n* = 200). Similarly, more pigs of WW class 2 (*P* < 0.001) and 3 (*P* = 0.073) were allocated to the CTRL (respectively *n* = 199 and *n* = 176) vs. the HIGH regime (respectively *n* = 143 and *n* = 153). The opposite (*P* = 0.003) was the case for piglets of WW class 4 (*n* = 149 for CTRL vs. *n* = 187 for HIGH). The differences in number of pigs per starter regime was a result of adhering to normal farm practices: animals were not allocated to different pens on the basis of their actual BW but on the basis of size (e.g. small, medium, or large). In addition, since classes were created post-hoc, each pen often consisted of a mixture of various WW classes rather than one class only. Nevertheless, pen variation was not significantly different across treatments (*P* > 0.05). Total post-weaning removal was 2.0%, with pigs in WW class 1 being removed in the highest quantity (6.8%, *P* < 0.001), compared to pigs of WW class 2, 3, and 4 (< 1.0%).

Table 3b shows the effect of WW class and starter regime on subsequent performance. Weaning weight class influenced pigs BW throughout the different stages of growth. Pigs of the lightest WW class (WW class 1), remained light throughout the different stages of production weighing almost 8.0 kg lighter at finisher compared to pigs weaned heavy (respectively 39.7 kg, SD = 12.3; and 47.5 kg, SD = 11.4). No difference (*P* > 0.05) in final weights (d 97) were observed between pigs from WW class 2 and 3. Neither starter regime nor the interaction between starter regime and WW class affected post-weaning performance (*P* > 0.05).

### Growth between birth and subsequent stages

**Fig. 1:**
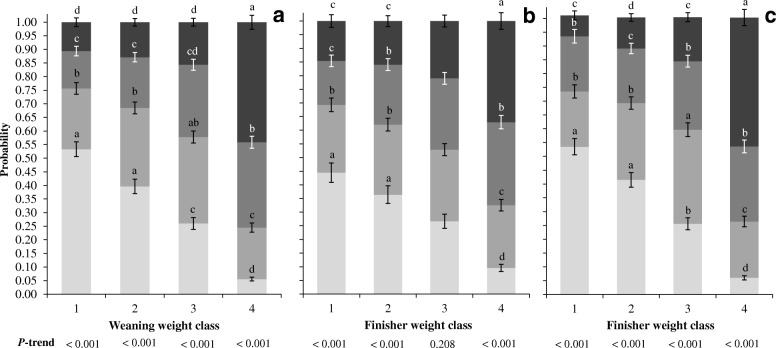
Piglet cumulative probability to change body weight (BW) class between birth (BiW) and weaning **a**, birth and finisher **b**, and weaning (WW) and finisher (**c**). Within batch, BW classes were created using percentiles (25%) resulting in 4 groups. Class 1 represents the lightest pig, class 4 the heaviest. Data is represented in probability ± SE. Different colours represent BW class, with respectively class 1 , class 2 , class 3 , and class 4 . The comparison is made between pigs of different BW classes (i.e. BiW or WW class) estimating their probability to end up in one of the final BW classes (i.e. weaning or finisher). Pigs were weighed within 12 h after birth (d 0), weaning (d 27.7, SD = 1.07), and finisher (d 98.8, SD = 0.937). ^a,b,c,d^ Within BW class (i.e. weaning, finisher) numbers with different superscripts differ significantly (*P* < 0.05)

Figure 1a and 1b show the cumulative probability of the various BiW classes to change class between birth and weaning, and birth and finisher respectively. The likelihood to end up light at weaning and finisher increased with decreasing BiW class. Birth weight class 1 piglets fed the HIGH regime had a higher likelihood (*P* < 0.001) to remain light (0.603, SD = 0.043 vs. 0.398, SD = 0.044) and a lower likelihood to end up heavy at finisher (0.055, SD = 0.010 vs. 0.103, SD = 0.016) than the same class piglets fed the CTRL regime.

Significant correlations were found between the different predictor variables assessed for each BiW class separate as shown in Additional file 3: Table S3. As expected BMI and PI were highly correlated for all BiW classes. Additional files 4 and 5: Figures S1 and S2 shows the effect of various morphometric characteristics on pig ability to change BW class from respectively birth to weaning and from birth to finisher. Apart from HL the majority of morphometric characteristics affected class change of especially BiW class 1 pigs. The effect of pre-weaning performance on pig ability to change BW class between birth and finisher is summarised in Additional file 6: Figure S3a. It is evident that for all BiW classes, piglet odds to end up light at finisher decreased with one unit increase in ADG (*P* < 0.001).

Table 4 shows the final multivariate regression models for the various BiW classes and the effect of different predictor variables on pre- and post-weaning ADG. The final multivariate regression model for BiW class 1 pigs showed that relative BiW (*P* = 0.026) and BMI (*P* = 0.003) were the most important factors for predicting the pre-weaning performance of BiW class 1 pigs, being positively associated with growth. It has to be noted however, that relative BiW (*P* < 0.001) was highly correlated (Additional file 3: Table S3) with other variables that were significant in the univariate model (Additional file 7: Table S4) such as BiW (*r* = 0.830), BiW:CC (*r* = 0.824) and HL:BiW (*r* = − 0.780). Although, significantly more (*P* = 0.003) BiW class 1 pigs remained light (class 1) at weaning (56.1%), compared to those that were able to increase class (43.9%), BiW class 1 pigs that were able to increase class pre-weaning, had a significant (*P* < 0.001) higher OR (95% CI); 5.11 [2.87, 9.10], 7.20 [3.59, 14.5], and 11.5 [2.53, 52.2] for respectively class 2–4, to end up heavy at finisher (FW class 3 and 4) than pigs that remained light at weaning (reference). However, the OR to end up heavy at finisher did not differ (*P* > 0.05) among BiW class 1 pigs that increased class (e.g. WW class 2, 3 or 4 piglets). The best fit multivariate model for post-weaning performance (Table 4) of BiW class 1 piglets consisted of only BiW:CC (*P* = 0.011) and WW (*P* = 0.001).

**Table 4:** Final multivariate models (coefficient and SE) of different predictor variables for pre- (d 0 to 28) and post-weaning ADG (d 28 to 99) for piglets from different body weight classes. Within batch, BW classes were created using percentiles (25%) resulting in 4 groups at birth (d 0) and weaning (d 27.7, SD = 1.07). Class 1 represents the lightest, class 4 the heaviest pigs. Morphometric measurements were taken within 12 h post-partum and pigs were weighed at birth, weaning and again at finisher (d 98.8; SD = 0.937)

Body weight class	Birth weight class								Weaning weight class			
Average daily gain, g/day	d 0–28				d 28–99				d 28–99			
	Class 1	Class 2	Class 3	Class 4	Class 1	Class 2	Class 3	Class 4	Class 1	Class 2	Class 3	Class 4
Predictor variable												
Birth weight, kg	–	–	–	54.5 (21.0)	–	–	–	–	–	–	–	–
Relative birth weight^1^	47.5 (21.1)	–	–	71.7 (27.1)	–	–	–	–	–	–	75.2 (33.9)	–
Snout to ear length, cm	–	–	–	12.1 (5.05)	–	–	–	–	–	–	–	–
Abdominal circumference, cm	–	–	3.55 (1.77)	–	–	–	–	–	–	5.89 (2.27)	–	–
Body mass index^2^, kg/m^2^	3.20 (1.06)	–	–	–	–	–	–	–	–	–	–	–
Ponderal index^3^, kg/m^3^	–	–	–	–	–	–	–	–	0.688 (0.319)	–	–	–
Birth weight: Cranial circumference, kg/cm	–	1729 (674)	–	–	2107 (824)	–	–	–	1128 (409)	–	–	1542 (581)
Gender^4^	–	11.5 (4.53)	–	–	–	–	–	–	–	−20.7 (8.59)	–	–
Weaning weight, kg	–	–	–	–	15.4 (4.50)	12.0 (4.24)	11.0 (3.76)	15.1 (3.42)	36.2 (6.57)	–	–	–
Pre-weaning ADG, g/day	–	–	–	–	–	–	–	–	–	−0.670 (0.261)	–	–

^1^Relative birth weight = (Birth weight piglet/ mean birth weight birth litter)

^2^Body mass index = birth weight (kg)/[crown rump length (m)]^2^

^3^Ponderal index = birth weight (kg)/[crown rump length (m)]^3^

^4^The coefficient reflects that of male, female was set as reference (0)

The principal cluster analysis of BiW class 1 piglets showed that two principal components had an Eigenvalue greater than 1. Together they explained 79.6% of the total variation: 59.4% by principal component 1 and 20.2% by principal component 2. Three clusters were formed; the description of the different clusters based on the variables used in the PCA are shown in Table 5. The majority of the piglets belonged to cluster 1 (44.2%), followed by cluster 2 (34.6%) and cluster 3 (21.2%) piglets. Cluster 2 piglets were the lightest at birth, relatively lighter compared to their average birth litter and differed significantly with respect to the various morphometric characteristics from cluster 1 and 3 piglets. While piglets of cluster 1 and 3 were born with a similar BiW, the differences in morphometric characteristics (i.e. HL, CRL, BMI, PI) suggest that piglets of cluster 1 were born proportionally long and thin compared to cluster 3 piglets. Pre-weaning mortality was significantly (*P* = 0.002) higher for cluster 2 piglets (34.1%) compared to cluster 1 (16.4%) and cluster 3 (21.5%) piglets. Piglets belonging to cluster 2 were weaned significantly (*P* < 0.001) lighter (5.75 kg, SD = 1.46) compared to cluster 1 and 3 piglets (respectively 6.11 kg, SD = 1.54 and 6.33 kg, SD = 1.56), however post-weaning performance was not significantly (*P* > 0.05) different among the different clusters.

**Table 5:** Cluster characteristics of birth weight class 1 piglets (smallest 25% at birth) clustered in different groups based on birth weight and various morphometric characteristics. Data are expressed as LSM ± SED^1^

Cluster (%)	1	2	3	SED	*P*-value
	44.2%	34.6%	21.2%		
Cluster characteristics					
Body weight, kg					
d 0	1.10^a^	0.835^b^	1.10^a^	0.002	< 0.001
Relative BiW^2^	0.807^a^	0.610^b^	0.794^a^	0.002	< 0.001
Morphometric characteristics					
Abdominal circumferences, cm	20.0^b^	17.9^a^	19.8^b^	0.051	< 0.001
Cranial circumferences, cm	20.0^b^	18.7^a^	20.0^b^	0.014	< 0.001
Snout to ear length, cm	9.64^a^	9.18^c^	9.47^b^	0.010	< 0.001
Crown rump length, cm	23.9^a^	21.5^b^	21.8^b^	0.021	< 0.001
Body mass index^3^, kg/m^2^	19.5^b^	18.0^c^	23.0^a^	0.032	< 0.001
Ponderal index^4^, kg/m^3^	81.4^b^	83.9^b^	106^a^	0.183	< 0.001
Birth weight: Cranial circumferences, kg/cm	0.0549^a^	0.0458^b^	0.0546^a^	0.0001	< 0.001
Snout to ear length: Birth weight, cm/ kg	8.84^b^	11.3^a^	8.74^b^	0.025	< 0.001

^a,b,c^Values with different superscripts differ significantly (*P* < 0.05)

^1^Pigs were weighed within 12 h post-partum (d 0)

^2^Relative birth weight = (Birth weight piglet/ mean birth weight birth litter)

^3^Body mass index = birth weight (kg)/[crown rump length (m)]^2^

^4^Ponderal index = birth weight (kg)/[crown rump length (m)]^3^

The majority of BiW class 2 pigs were able to increase class between BiW and WW (44.9%) and BiW and FW (47.9%) compared to those that remained (respectively 31.3 and 25.3%) or decreased BW class (respectively 23.9 and 26.7%). Those that decreased class pre-weaning, had a significant (*P* < 0.001) lower OR (0.483 [0.273, 0.854]) to end up heavier at finisher (class 4) compared to BiW class 2 pigs that remained or increased class, respectively class 2 (reference), 3 (1.38 [0.812, 2.36]) and 4 (3.28 [1.70, 6.31]). On the other hand, most pigs of BiW class 3 decreased class between BiW and WW (40.2%) and BiW and FW (42.5%). Consequentially, BiW class 2 and 3 piglets had a similar probability (*P* > 0.05) to end up in WW class 3, FW class 2, and FW class 3 as shown in Fig. 1a and b respectively. Gender (*P* = 0.012) and BiW: CC (*P* = 0.012) were able to predict pre-weaning performance of BiW class 2 pigs (Table 4) whereas, WW was the sole variable in the multivariate model and was positively associated with post-weaning performance for BiW class 2 to 4 pigs.

### Growth between weaning and finisher

Weaning weight class 1 piglets had the highest likelihood (*P* < 0.05) to remain light (class 1 and 2) at finisher and were less likely to end up heavy at finisher (class 4) compared to WW class 2 to 4 piglets (Fig. 1c). Although significantly more WW class 1 piglets (54.1%) remained light at finisher (*P* = 0.047), 45.1% were able to increase class. Additional file 6: Figure S3b shows the effect of pre-weaning ADG on piglet ability to change BW class between weaning and finisher. Pre-weaning ADG only significantly influenced class change for piglets weaned below average (class 1 and 2). Class change between WW and FW was also significantly affected by the different morphometric characterises (see Additional file 8: Figure S4) and mostly affected WW class 1 piglets.

The majority (*P* < 0.001) of piglets weaned light (WW class 1) were born light (48.1%), the rest was weaned light but born heavier: BiW class 2 (23.3%), 3 (15.7%) and 4 (12.8%). Piglets born heavier (BiW class 2 to 4) but weaned light had a significantly (*P* < 0.001) higher OR (respectively, 3.17 [1.80, 5.58], 3.60, [1.87, 6.92], and 3.67, [1.80, 7.48]) to end up heavy at finisher (class 4) compared to piglets of BiW class 1 (reference). The multivariate regression model (Table 4) including piglets that were alive at finisher (d 99), suggests that BiW:CC (*P* < 0.001), PI (*P* = 0.037), and WW (*P* < 0.001) were the best predictors for post-weaning performance, being positively associated with growth of WW class 1 piglets. It has to be noted that BiW:CC was positively correlated to BiW (*r* = 0.983), relative BiW (*r* = 0.856) and various morphometric characteristics (*r* > 0.70) such as AC, CC, BMI, and HL:BiW (Additional file 9: Table S5) that appeared to be significant in the univariate analysis (Additional file 10: Table S6).

The description of the cluster analysis for WW class 1 piglets based on the variables used in the PCA is shown in Table 6. Three principal components had an Eigenvalue greater than 1 and together explained 87.4% of the total variation: 57.9% principal component 1, 16.0% principal component 2, and 13.5% principal component 3. The majority of the piglets belonged to cluster 2 (61.9%), followed by cluster 3 (22.0%), and cluster 1 (16.1%) piglets. Cluster 1 piglets were born and weaned significantly lighter and differed to cluster 2 and 3 piglets with respect to various morphometric characteristics (e.g. CC, HL, BIW:CC and HL:BiW). Cluster 3 piglets had the highest BiW, had a significantly (*P* < 0.001) higher BMI, PI, AC, and BiW:CC compared to cluster 2 piglets, but were weaned with a similar BW (*P* > 0.05). Post-weaning mortality (d 28 to 61) did not differ between the different clusters (*P* > 0.05); with 10.9, 5.21, and 6.67% for respectively cluster 1, 2, and 3. However, cluster 1 piglets remained the lightest (*P* < 0.001; 35.3 kg, SD = 6.73) post-weaning, weighing 5 to 6.8 kg lighter at finisher compared to cluster 2 and 3 piglets (respectively 40.2 kg, SD = 7.21 and 42.1 kg, SD = 6.33). Although, cluster 3 piglets had a significantly lower pre-weaning ADG (*P* = 0.027) than cluster 2 piglets, at finisher cluster 3 piglets tended (*P* = 0.073) to weigh almost 2 kg heavier at finisher compared to cluster 2 piglets.

**Table 6:** Cluster characteristics of weaning weight class 1 piglets (smallest 25% at weaning) clustered in different groups based on birth weight, various morphometric characteristics, and pre-weaning growth. Data are expressed as LSM ± SED^1^

Cluster (%)	1	2	3	SED	*P*-value
	16.1%	61.9%	22.0%		
Cluster characteristics					
Body weight, kg					
d 0	1.13^c^	1.26^b^	1.46^a^	0.007	< 0.001
Relative BiW^2^	0.795^c^	0.887^b^	0.988^a^	0.004	< 0.001
d 28	3.91^b^	5.53^a^	5.54^a^	0.010	< 0.001
Morphometric characteristics					
Abdominal circumferences, cm	20.0^b^	20.7^b^	22.0^a^	0.054	< 0.001
Cranial circumferences, cm	20.0^c^	20.5^b^	21.3^a^	0.033	< 0.001
Snout to ear length, cm	9.51^c^	9.75^b^	9.92^a^	0.015	< 0.001
Crown rump length, cm	23.3^b^	24.5^a^	23.8^b^	0.049	< 0.001
Body mass index^3^, kg/m^2^	20.2^b^	20.5^b^	25.6^a^	0.058	< 0.001
Ponderal index^4^, kg/m^3^	86.5^b^	84.2^b^	108^a^	0.245	< 0.001
Birth weight: Cranial circumferences, kg/cm	0.0553^c^	0.0607^b^	0.0683^a^	0.0003	< 0.001
Snout to ear length: Birth weight, cm/ kg	9.23^c^	8.23^b^	7.09^a^	0.045	< 0.001
Average daily gain, g/day					
d 0–28	99.5^c^	154^a^	147^b^	0.459	< 0.001

^a,b,c^Values with different superscripts differ significantly (*P* < 0.05)

^1^Pigs were weighed within 12 h post-partum (d 0) and at weaning (d 27.7, SD = 1.07)

^2^Relative birth weight = (Birth weight piglet/ mean birth weight birth litter)

^3^Body mass index = birth weight (kg)/[crown rump length (m)]^2^

^4^Ponderal index = birth weight (kg)/[crown rump length (m)]^3^

The majority of class 2 piglets (49.0%) were able to increase class at finisher, whereas the majority (41.6%) of WW class 3 piglets decreased class between WW and FW. As a result, piglets of WW class 2 had a higher probability to finish in FW class 3 compared to WW class 3 piglets (respectively 0.337, SD = 0.025 and 0.247, SD = 0.022, Fig. 1c). Abdominal circumferences (*P* = 0.010), gender (*P* = 0.017), and pre-weaning ADG (*P* = 0.011) were significant in the final model for WW class 2 piglets (Table 4). None of the morphometric characteristics were significant in the univariate models for WW class 3 piglets and only relative BiW appeared to be significant (*P* = 0.028). The final multifactorial model of WW class 4 piglets consisted of BiW:CC (*P* = 0.009) only.

## Discussion

Maximising sow reproductive potential via genetic selection for the total number of piglets born or weaned has resulted in increased litter sizes and thus the number of piglets weaned per sow per year [22]. Although the number of piglets produced per sow per year is an important economic trait, as a result of limitations in the uterine capacity and maternal resources larger litter sizes increase the proportion of piglets born light [23–26], the number of intrauterine growth restricted (IUGR) piglets [26] and consequently increase within-litter variation [23]. To ensure that piglet quality and welfare is not compromised, pig producers are increasingly challenged to keep lightweight piglets alive and to improve weaning weight, minimising batch inefficiency. Knowing which piglets would benefit from intervention strategies at different stages of growth is important, as some lightweight pigs may be able to perform better without intervention, minimising variable growth rates within a group and possible economic losses. Although, recent research suggests that body shape at birth (e.g. BMI, AC) and not BiW were able to predict postnatal growth of piglets born light [9], research assessing the effect of body shape at birth together with well-known predictor variables such as BiW, WW and pre-weaning ADG on subsequent performance under commercial conditions is scarce. Our work is the first attempt that addresses these issues up to finishing stage. In addition, we have applied novel statistical methodologies to answer these questions. This allows us to make predictions about the effects of morphometric measurements on subsequent performance.

Furthermore, we investigated piglet ability for improved performance when given access to a nutrient enriched regime, i.e. with a higher EAA:NE ratio, at weaning. Improved starter regimes tailored on the basis of lightweight piglet requirements rather than the average piglet, have been shown to be effective in improving post-weaning performance for lightweight pigs [10, 11]. The low feed intake [12, 13], the lower serum concentrations of EAA [5] and possible higher protein turnover [14], suggest that lightweight piglets may benefit from an EAA enriched diet. Piglets weaned light appear to have an immature digestive system [27, 28] and a higher epithelial cell turnover [29], and therefore may benefit from an increased supply of threonine [30] and methionine [31]. Their lower ghrelin expression [32] and serotonin concentrations [33] suggest that an increased supplementation of tryptophan may stimulate appetite [30].

The results of our study did not support the hypothesis that piglets weaned light would improve performance when having access to a regime higher in EAA. In the nutrient enriched regimes lysine was increased by 20% with the other EAA being balanced to lysine, ensuring the appropriate ratios were maintained [18]. Reasons for the lack of effect could be a result of: 1) lightweight pigs actually not having the hypothesised higher EAA requirements post-weaning [15], 2) lightweight piglets not having access to enough energy to use the extra EAA supplied, and 3) an absence of a specific response to the supplemented AAs, including their ratios to lysine. For example, there are suggestions that individual AA, such as arginine and glutamine [34], may enhance the growth of lightweight piglets. Although studies looking at infants that were born extremely light suggest that a more concentrated diet with increased levels of energy and AA accelerate weight gain [35], others [12] who have hypothesised that piglets weaned light would benefit from a more nutrient dense diet (i.e. different energy levels), were also unable to find a positive effect on the post-weaning performance.

Birth [1, 2, 36] and weaning weight [3–6, 36] have been identified as predictors of post-weaning growth. Although, piglets born light (< 1.00 kg) have a higher mortality rate [8], a higher feed conversion ratio [13] and need more time to reach market weight [24], one should discriminate between piglets that have been born light for gestational age e.g. ‘proportionally small’ [26] and piglets that have suffered from growth restriction in utero. Different outcomes may be expected, with IUGR piglets believed to remain stunted throughout life [37]. In addition, the severity of IUGR might vary between pigs and is suggested to be dependent on the stage of gestation and duration; the longer the period of growth restriction in utero the lesser the ability to recover post-partum [37].

Although piglets born light have a higher likelihood to remain light at weaning and finisher, our results suggest that not all lightweight pigs are the same and that some are actually able to do better than others. This is supported by the following: 1) pre-weaning ADG and not BiW, was highly correlated with WW, 2) BW class change between birth and subsequent stages was under the influence of various morphometric characteristics, and 3) the multivariate analysis showed that relative BiW and BMI were positively associated with pre-weaning growth for BiW class 1 piglets rather than BiW per se. Douglas et al. [9] have put forward several reasons why such morphometric measurements may be better predictors of postnatal performance. The positive association between BMI and pre-weaning ADG may be a result of: 1) differences in surface area: volume ratio [38] influencing metabolic rate or 2) differences in the amount of maternal resources acquired during gestation important for development [9, 39, 40]. Relative birth weight, on the other hand, may suggest that the lightest piglet of the litter might have been at a competitive disadvantage for colostrum intake [41].

In contrast to what has been previously found [9], post-weaning ADG for BiW class 1 pigs was positively associated with WW and head shape at birth (i.e. BiW:CC), with the absence of WW in the previous study [9] most likely have contributed to the differences seen. The positive association between BiW:CC and post-weaning performance suggests that pigs with a larger head size in relation to BiW (low BiW:CC) have an impaired post-weaning performance. The dolphin-like forehead, the adaptive brain sparing effect as a result of placental insufficiency, has been used for the identification of IUGR piglets [8]: piglets that might not be able to display normal growth and remain stunted throughout life [38]. Also in our study BiW:CC discriminated between piglets that suffered from a certain degree of IUGR. On the other hand, these findings also emphasize the importance of weaning weight: a good start is essential and increasing BW class pre-weaning has been shown beneficial for subsequent performance. Weaning weight does not only influence subsequent performance [11–13, 36], with a higher likelihood for a slower post-weaning growth rate for WW class 1 (bottom 12.5% at weaning) compared to BiW class 1 pigs (bottom 12.5% at birth) [3], but is also an important factor influencing disease risks [42], post-weaning mortality [36] and batch efficiency in all-in-all-out systems, especially for pig enterprises from the bottom quartile [43]. This suggests that the emphasis should be on the pre-weaning management [44–48] for improving the performance of piglets born light. At the same time our results point towards which piglets are most likely to benefit from such sometimes time consuming and expensive strategies.

There are several reasons why a piglet born heavy might end up light at weaning, such as: direct and indirect competition for milk intake [45] and sickness. In our experiment 51.9% of piglets born heavy (e.g. BiW class 2 to 4) fell into this category. However, such piglets were still at an advantage for compensatory growth post-weaning, having a higher OR to end up heavy at finisher, compared to piglets born and weaned light. Compensatory growth after a period of stunting has previously been shown for piglets born heavier once having access to an better quality starter regime [49]. However, differences in pre-weaning nutrient intake might have set appetite during subsequent stages [50] and therefore piglets with a poor pre-weaning ADG achieve a lower growth potential than similar sized piglets with a greater pre-weaning ADG.

In addition, the multivariate and cluster analysis emphasised that not all piglets weaned light are the same: the distinction between piglets weaned light that can or cannot exhibit compensatory growth was not under the influence of BiW only. Our data suggest that piglets born and weaned light and born disproportional (cluster 1), were unable to improve performance under the commercial conditions of our experiment. Piglets of cluster 2 and 3, on the other hand, were weaned relatively heavier but differed from one another with respect to body shape at birth, with cluster 3 piglets having a greater post-weaning growth than cluster 2 piglets that were born lighter and relatively disproportional. The multivariate analysis for WW class 1 piglets, on the other hand, showed that post-weaning performance was not under the influence of BiW, but similarly to BiW class 1 piglets were positively associated with WW, and several morphometric characteristics i.e. PI and head shape at birth (BiW:CC). These morphometric characteristics [8, 38] may differentiate between pigs that have suffered from a certain degree of IUGR as discussed previously. Piglets that are born disproportional (e.g. low BiW:CC and PI) and weaned light may benefit from specialised strategies post-weaning. However, more research is necessary to determine whether these piglets can compensate when having access to an improved post-weaning environment or whether they remain stunted.

## Conclusions

This study suggests that a subset of piglets born lightweight are able to show compensatory growth. These are piglets that are characterised by a higher BMI and a higher relative birth weight, the relatively bigger piglets of the litter. Treating all lightweight pigs the same pre-weaning might explain why management strategies commonly applied are able to induce an improved performance, but are less likely to reduce litter CV. Piglets that are born long and thin (low BMI), on the other hand, would most likely benefit from pre-weaning intervention strategies. Post-weaning strategies should focus on pigs that are born disproportional (low PI and low BiW:CC) and on those weaned light. In other words, the results of the present study suggest that pig producers should discriminate between pigs that are weaned light on the basis of their birth characteristics to better target the often costly and time consuming intervention strategies, for example by ear-tagging the affected piglets at birth. In addition, researchers assessing the effect of post-weaning strategies on piglets weaned light should take caution when selecting piglets on the basis of weaning weight only, as piglet shape at birth might influence the experimental outcomes. In this study we were unable to demonstrate any benefits arising from the nutritional manipulation of the nursery regime on the lightweight piglets; all classes of lightweight piglets were unable to improve post-weaning performance when having access to the nutrient enriched regime (higher EAA:NE), which may suggest that lightweight piglets do not have higher EAA requirements.

## Additional files

Additional file 1: **Table S1.** Summary of final models used after removal of nonsignificant covariates. (DOCX 35 kb)

Additional file 2: **Table S2.** Pre- and post-weaning production characteristics. (DOCX 34.2 kb)

Additional file 3: **Table S3.** Rank correlations between predictor variables for piglets of a different birth weight (BiW) class. (DOCX 43 kb)

Additional file 4: **Figure S1.** Effect of various morphometric characteristics on pig ability (log odds ± SE) to change BW class between birth (BiW) and weaning (WW). (DOCX 205 kb)

Additional file 5: **Figure S2.** Effect of various morphometric characteristics on pig ability (log odds ± SE) to change BW class between birth (BiW) and finisher (FW). (DOCX 213 kb)

Additional file 6: **Figure S3.** Effect of pre-weaning ADG (d 0 to 28) on pig ability (log odds, SE) to change BW class between birth and finisher (A) and weaning and finisher (B). (DOCX 95 kb)

Additional file 7: **Table S4.** Statistical significance (*P* - value) of the different predictor variables fitted in the univariate models for piglets of a different birth weight (BiW) class for ADG (g/d) between d 0 to 28 and d 28 to 99. (DOCX 36 kb)

Additional file 8: **Figure S4.** Effect of various morphometric characteristics on pig ability (log odds ± SE) to change BW class between weaning (WW) and finisher (FW). (DOCX 232 kb)

Additional file 9: **Table S5.** Rank correlations between predictor variables for piglets of a different weaning weight (WW) class. (DOCX 42.8 kb)

Additional file 10: **Table S6.** Statistical significance (*P* - value) of the different predictor variables fitted in the univariate models for piglets of a different weaning weight (WW) class for ADG (g/d) between d 28 and 99. (DOCX 35 kb)
