# Supplementary material for: Once small always small? To what extent morphometric characteristics and post-weaning starter regime affect pig lifetime growth performance
Source: Porcine Health Manag. 2018 Jul 23;4:21. doi: 10.1186/s40813-018-0098-1 (PMC6055348; doi:10.1186/s40813-018-0098-1)
Supplement: Supplementary file 1 — Table S1. Summary of final models used after removal of nonsignificant covariates. (DOCX 35 kb) [file 40813_2018_98_MOESM1_ESM.docx]

**Table S1**

Summary of final models used after removal of nonsignificant covariates. Within batch, birth (BiW), weaning (WW), finisher (FW) weight classes were created retrospectively using percentiles resulting in 4 (25%) classes. At weaning pens were randomly allocated to one of the starter regimes: control (CTRL) vs. nutrient enriched starter regime (HIGH).

| **Objective** | **Parameter analysed** | **Unit** | **Model type** | **Fixed effects** | **Covariates** | **Random Effects** | **Weight** |
| --- | --- | --- | --- | --- | --- | --- | --- |
| **1)** Effect of BW class^1^ and starter regime on absolute performance | Birth weight | Piglet | PROC MIXED | BiW class, Gender | Total born, Batch | Foster sow nested within Batch, VC^3^ | - |
|  | Pre-weaning performance |  |  | BW class^1^, Gender, Starter regime,  BW class*Starter regime | Litter size^2^, Foster parity, Age, Batch |  | - |
|  | Post-weaning performance |  |  |  | Age, Batch,  Weaning weight^4,^ Pen variation^5^ | Room*pen nested within Batch, VC^3^ | - |
|  | Post weaning performance | Pen mean | PROC MIXED | Starter regime | Batch, Pen variation^5^, Gender | Room nested within Batch, VC^3^ | Group size^2^ |
| **2)** Effect of BW class^1^ and starter regime on class change | Final BW class (e.g. WW or FW class) 1, 2, 3, or 4^6^ | Piglet | PROC LOGISTIC, descending | BW class^1,7^, Starter regime^7^, BW class*Starter regime | Gender, Age | - | - |
| **3)** Effect of pigs characteristics on class change | Final BW class (e.g. WW or FW class) 1, 2, 3, or 4^6^ | Piglet^8^ | PROC LOGISTIC, descending | Pre-weaning ADG or various morphometric characteristics | Gender, Age | - | - |

^1^ BW (body weight) classes were BiW and WW class

^2^ Pre-weaning litter size/ group size post-weaning = [(total time (h) piglets reside within litter/ pen)/24 h]/ total period in d

^3^ Random effect type variance components

^4^ Weaning weight was only inserted in the model assessing the effect on BiW class on post-weaning performance.

^5^ Pen variation based on WW class representing the average BW class per pen. The latter, was only inserted in the model assessing the effect of WW class on post-weaning performance and the model with pen mean as experimental unit.

^6^ The response variable (final BW class) was formatted to enable to estimation of class change for the intermediate classes

^7^ The reference for BW class (e.g. BiW or WW class) was set to the final BW to be tested. The reference for starter regime was the CTRL regime.

^8^ The model was performed for each BW class separate (i.e. class 1, 2, 3, or 4), omitting BW class as independent variable
