# Supplementary material for: Once small always small? To what extent morphometric characteristics and post-weaning starter regime affect pig lifetime growth performance
Source: Porcine Health Manag. 2018 Jul 23;4:21. doi: 10.1186/s40813-018-0098-1 (PMC6055348; doi:10.1186/s40813-018-0098-1)
Supplement: Supplementary file 2 — Table S2. Pre- and post-weaning production characteristics. (DOCX 34.2 kb) [file 40813_2018_98_MOESM2_ESM.docx]

**Table S2**

Pre- and post-weaning production characteristics. All newly born piglets from 137 litters originating from 8 consecutive farrowing batches were followed from birth to weaning (WW, d 28) and finishing (d 99) following normal farm practises.

| **Characteristics** |  | **Range** |
| --- | --- | --- |
| **At birth** |  |  |
| Number of piglets born alive, total | 1487 | - |
| Males, % | 57 | - |
| Litter size | 12.6 | 2 to 19 |
| Still born | 92 (5.4%) | - |
| Mummified | 40 (2.3%) | - |
| Crushed within 12h post-partum | 23 (1.3%) | - |
| Birth weight, kg | 1.47 (SD = 0.340) | 0.454 to 2.45 |
| **Litter CV**^1^ |  | - |
| Birth | 18.6 (SD = 5.63) |  |
| After cross-fostering | 12.2 (SD = 4.88) |  |
| **Pre-weaning** |  |  |
| Number of piglets cross-fostered, % | 57.7 | - |
| Pre-weaning mortality, %^1^ | 8.4 | - |
| Weaning weight, kg^2^ | 7.18 (SD = 1.60) | 2.18 to 12.4 |
| **Body weight post-weaning**^2^ |  |  |
| d 48, kg | 14.7 (SD = 2.78) | 4.93 to 25.4 |
| d 61, kg | 22.7 (SD = 3.98) | 9.40 to 36.6 |
| d 74, kg^3^ | 32.6 (SD = 2.91) | 23.8 to 38.8 |
| d 99, kg | 44.9 (SD = 6.39) | 21.6 to 67.4 |

^1^ Based on the piglets that were alive at the first processing (< 12 h post-partum).

^2^ Pigs were weighed at weaning (d 27.7; SD = 1.07), 3 weeks (48.0; SD = 0.887), 5 weeks (d 61.5; SD = 1.17), and 10 weeks post-weaning (d 98.8, SD = 0.938).

^3^ Pigs that were sold earlier (d 74.8, SD = 1.93)
