# Supplementary material for: Once small always small? To what extent morphometric characteristics and post-weaning starter regime affect pig lifetime growth performance
Source: Porcine Health Manag. 2018 Jul 23;4:21. doi: 10.1186/s40813-018-0098-1 (PMC6055348; doi:10.1186/s40813-018-0098-1)
Supplement: Supplementary file 3 — Table S3. Rank correlations between predictor variables for piglets of a different birth weight (BiW) class. (DOCX 43 kb) [file 40813_2018_98_MOESM3_ESM.docx]

**Table S3**

Rank correlations between predictor variables for piglets of a different birth weight (BiW) class. Within batch, BiW classes were created retrospectively using percentiles resulting in 4 (25%) classes: class 1 represents the lightest pig, class 4 the heaviest. Numbers in bold were variables that were considered highly correlated (*r* > +/- 0.70).^1,2^

**BiW class 1**

| Predictor variable | BiW | Rel BiW | WW | ADG | CRL | HL | AC | CC | BMI | PI | BiW: CC | HL: BiW |
| --- | --- | --- | --- | --- | --- | --- | --- | --- | --- | --- | --- | --- |
| Birth weight (BiW), kg | - |  |  |  |  |  |  |  |  |  |  |  |
| Relative Birth weight (Rel BiW)^3^ | **0.830** | - |  |  |  |  |  |  |  |  |  |  |
| Weaning weight (WW), kg | 0.431 | 0.367 | - |  |  |  |  |  |  |  |  |  |
| Pre-weaning ADG (ADG), g/day | 0.327 | 0.291 | **0.982** | - |  |  |  |  |  |  |  |  |
| Crown to rump length (CRL), cm | **0.728** | 0.650 | 0.230 | 0.171 | - |  |  |  |  |  |  |  |
| Snout to ear length (HL), cm | 0.442 | 0.409 | 0.083 | *ns* | 0.396 | - |  |  |  |  |  |  |
| Abdominal circumference (AC), cm | **0.701** | 0.552 | 0.194 | 0.131 | 0.557 | 0.313 | - |  |  |  |  |  |
| Cranial circumference (CC), cm | **0.782** | 0.614 | 0.322 | 0.241 | 0.568 | 0.340 | 0.643 | - |  |  |  |  |
| Body mass index^4^(BMI), kg/m^2^ | 0.640 | 0.492 | 0.328 | 0.252 | *ns* | 0.213 | 0.411 | 0.509 | - |  |  |  |
| Ponderal index^5^ (PI), kg/m^3^ | 0.210 | 0.126 | 0.168 | 0.132 | -0.500 | *ns* | *ns* | 0.173 | **0.883** | - |  |  |
| BiW: CC, kg/cm | **0.973** | **0.824** | 0.415 | 0.316 | **0.715** | 0.436 | 0.651 | 0.620 | 0.620 | 0.200 | - |  |
| HL: BiW, cm/kg | **-0.935** | **-0.780** | -0.429 | -0.335 | -0.682 | -0.195 | -0.676 | **-0.745** | -0.625 | -0.222 | **-0.916** | - |

**BiW class 2**

| Predictor variable | BiW | Rel BiW | WW | ADG | CRL | HL | AC | CC | BMI | PI | BiW: CC | HL: BiW |
| --- | --- | --- | --- | --- | --- | --- | --- | --- | --- | --- | --- | --- |
| Birth weight (BiW), kg | - |  |  |  |  |  |  |  |  |  |  |  |
| Relative Birth weight (Rel BiW)^3^ | 0.302 | - |  |  |  |  |  |  |  |  |  |  |
| Weaning weight (WW), kg | 0.192 | 0.002 | - |  |  |  |  |  |  |  |  |  |
| Pre-weaning ADG (ADG), g/day | 0.131 | 0.005 | **0.981** | - |  |  |  |  |  |  |  |  |
| Crown to rump length (CRL), cm | 0.276 | 0.150 | *ns* | *ns* | - |  |  |  |  |  |  |  |
| Snout to ear length (HL), cm | 0.224 | 0.124 | *ns* | *ns* | 0.171 | - |  |  |  |  |  |  |
| Abdominal circumference (AC), cm | 0.246 | 0.123 | *ns* | *ns* | 0.228 | *ns* | - |  |  |  |  |  |
| Cranial circumference (CC), cm | 0.384 | *ns* | 0.123 | *ns* | *ns* | *ns* | 0.169 | - |  |  |  |  |
| Body mass index^4^(BMI), kg/m^2^ | 0.263 | *ns* | *ns* | *ns* | **-0.846** | *ns* | *ns* | 0.117 | - |  |  |  |
| Ponderal index^5^ (PI), kg/m^3^ | *ns* | *ns* | *ns* | *ns* | **-0.921** | *ns* | -0.140 | *ns* | **0.983** | - |  |  |
| BiW: CC, kg/cm | **0.820** | 0.300 | 0.121 | *ns* | 0.236 | 0.180 | 0.155 | -0.212 | 0.202 | *ns* | - |  |
| HL: BiW, cm/kg | -0.635 | -0.148 | *ns* | *ns* | *ns* | 0.608 | -0.155 | -0.245 | -0.252 | -0.140 | -0.521 | - |

**BiW class 3**

| Predictor variable | BiW | Rel BiW | WW | ADG | CRL | HL | AC | CC | BMI | PI | BiW: CC | HL: BiW |
| --- | --- | --- | --- | --- | --- | --- | --- | --- | --- | --- | --- | --- |
| Birth weight (BiW), kg | - |  |  |  |  |  |  |  |  |  |  |  |
| Relative Birth weight (Rel BiW)^3^ | 0.120 | - |  |  |  |  |  |  |  |  |  |  |
| Weaning weight (WW), kg | 0.123 | *ns* | - |  |  |  |  |  |  |  |  |  |
| Pre-weaning ADG (ADG), g/day | *ns* | *ns* | **0.975** | - |  |  |  |  |  |  |  |  |
| Crown to rump length (CRL), cm | 0.352 | *ns* | *ns* | *ns* | - |  |  |  |  |  |  |  |
| Snout to ear length (HL), cm | 0.240 | *ns* | *ns* | *ns* | 0.186 | - |  |  |  |  |  |  |
| Abdominal circumference (AC), cm | 0.191 | 0.137 | 0.130 | *ns* | 0.145 | *ns* | - |  |  |  |  |  |
| Cranial circumference (CC), cm | 0.398 | *ns* | *ns* | *ns* | *ns* | 0.162 | 0.179 | - |  |  |  |  |
| Body mass index^4^(BMI), kg/m^2^ | 0.136 | *ns* | *ns* | *ns* | **-0.872** | *ns* | *ns* | *ns* | - |  |  |  |
| Ponderal index^5^ (PI), kg/m^3^ | *ns* | *ns* | *ns* | *ns* | **-0.931** | -0.105 | *ns* | *ns* | **0.986** | - |  |  |
| BiW: CC, kg/cm | **0.786** | 0.140 | *ns* | *ns* | 0.309 | 0.146 | *ns* | -0.254 | *ns* | *ns* | - |  |
| HL: BiW, cm/kg | -0.626 | *ns* | *ns* | *ns* | -0.141 | 0.605 | -0.109 | -0.194 | -0.163 | *ns* | -0.528 | - |

**BiW class 4**

| Predictor variable | BiW | Rel BiW | WW | ADG | CRL | HL | AC | CC | BMI | PI | BiW: CC | HL: BiW |
| --- | --- | --- | --- | --- | --- | --- | --- | --- | --- | --- | --- | --- |
| Birth weight (BiW), kg | - |  |  |  |  |  |  |  |  |  |  |  |
| Relative Birth weight (Rel BiW)^3^ | 0.251 | - |  |  |  |  |  |  |  |  |  |  |
| Weaning weight (WW), kg | 0.313 | 0.193 | - |  |  |  |  |  |  |  |  |  |
| Pre-weaning ADG (ADG), g/day | 0.211 | 0.185 | **0.984** | - |  |  |  |  |  |  |  |  |
| Crown to rump length (CRL), cm | 0.348 | *ns* | 0.227 | 0.179 | - |  |  |  |  |  |  |  |
| Snout to ear length (HL), cm | 0.290 | *ns* | 0.160 | 0.148 | 0.208 | - |  |  |  |  |  |  |
| Abdominal circumference (AC), cm | 0.337 | 0.237 | 0.206 | 0.165 | 0.151 | *ns* | - |  |  |  |  |  |
| Cranial circumference (CC), cm | 0.536 | *ns* | 0.155 | 0.105 | 0.150 | 0.168 | 0.259 | - |  |  |  |  |
| Body mass index^4^(BMI), kg/m^2^ | 0.354 | 0.146 | *ns* | *ns* | -0.746 | *ns* | *ns* | 0.216 | - |  |  |  |
| Ponderal index^5^ (PI), kg/m^3^ | 0.121 | *ns* | *ns* | *ns* | **-0.875** | *ns* | *ns* | *ns* | **0.970** | - |  |  |
| BiW: CC, kg/cm | **0.884** | 0.266 | 0.280 | 0.187 | 0.332 | 0.251 | 0.249 | *ns* | 0.296 | *ns* | - |  |
| HL: BiW, cm/kg | **-0.784** | -0.248 | -0.199 | -0.107 | -0.201 | 0.357 | -0.302 | -0.412 | -0.354 | -0.178 | -0.699 | - |

^1^ Pearson correlation test was used to estimate correlations between continuous variables that were normally distributed. Variables with a high correlation (*r* ≥ 0.70) are in bold. Morphometric measurements were taken within 12 h post-partum, pigs were weighed at birth (d 0) and at weaning (d 27.7; SD = 1.07).

^2^ *ns* = not significant (*P* > 0.05)

^3^ Relative birth weight = (Birth weight piglet/ mean birth weight birth litter)

^4^ Body mass index = birth weight (kg)/[crown rump length (m)]^2^

^5^ Ponderal index = birth weight (kg)/[crown rump length (m)]^3^
