# Supplementary material for: Once small always small? To what extent morphometric characteristics and post-weaning starter regime affect pig lifetime growth performance
Source: Porcine Health Manag. 2018 Jul 23;4:21. doi: 10.1186/s40813-018-0098-1 (PMC6055348; doi:10.1186/s40813-018-0098-1)
Supplement: Supplementary file 5 — Figure S2. Effect of various morphometric characteristics on pig ability (log odds ± SE) to change BW class between birth (BiW) and finisher (FW). (DOCX 213 kb) [file 40813_2018_98_MOESM5_ESM.docx]

**

**

*■ µ 22.7*

*± 1.69*

*■ µ 24.8*

*± 1.38*

*■ µ 25.9*

*± 1.49*

*■ µ 27.1*

*± 1.60*

*■ µ 19.3*

*± 1.83*

*■ µ 21.8*

*± 1.44*

*■ µ 22.8*

*± 1.47*

*■ µ 24.2*

*± 1.56*

*

**

**

**

**

**

**

**

*

**

**

**

**

*

**

**

**

*

*■ µ 10.0 ± 0.538*

*■ µ 9.46*

*± 0.571*

*■ µ 10.2*

*± 0.537*

*■ µ 10.4*

*± 0.570*

*■ µ 19.6*

*± 1.07*

*■ µ 21.0*

*± 0.741*

*■ µ 21.6*

*± 0.743*

**

*■ µ 22.6*

*± 0.892*

**

*

**

*

**

**

*

**

**

*

**

**

*

*

**

*

*

**

*■ µ 92.1*

*± 14.9*

*■ µ 87.6*

*± 13.8*

*■ µ 91.5*

*± 14.6*

*■ µ 96.0*

*± 16.6*

*■ µ 19.8*

*± 2.64*

*■ µ 22.5*

*± 2.46*

*■ µ 23.7*

*± 2.58*

*■ µ 25.8*

*± 3.12*

**

**

**

*

*

**

**

*

*

**

**

**

**

*■ µ 0.0519*

*± 0.00772*

*■ µ 0.0653*

*± 0.00367*

*■ µ 0.0731*

*± 0.00374*

*■ µ 0.0833*

*± 0.00604*

*■ µ 7.27*

*± 0.538*

*■ µ 9.58*

*± 1.91*

*■ µ 6.44*

*± 0.424*

*■ µ 5.57*

*± 0.476*

4

3

2

3

4

2

1

1

**Figure S2**

Effect of various morphometric characteristics on pig ability (log odds ± SE) to change BW class between birth (BiW) and finisher (FW). Within batch, BW classes were created using percentiles (25%) resulting in 4 groups. Class 1 represents the lightest pig, class 4 the heaviest. The different colours represent BiW class, with respectively class 1 **■**, class 2 **■**, class 3 **■**, and class 4 **■**. Coefficients were estimated for each BiW class separate. Morphometric measurements were taken within 12 h post-partum, pigs were weighed at birth (d 0) and again at finisher (d 98.8; SD = 0.937). The µ ± SED on the x-axis represent the average of the characteristic of interest for each BiW class. ** (*P* < 0.05), * (*P* < 0.10)
