# Supplementary material for: Once small always small? To what extent morphometric characteristics and post-weaning starter regime affect pig lifetime growth performance
Source: Porcine Health Manag. 2018 Jul 23;4:21. doi: 10.1186/s40813-018-0098-1 (PMC6055348; doi:10.1186/s40813-018-0098-1)
Supplement: Supplementary file 6 — Figure S3. Effect of pre-weaning ADG (d 0 to 28) on pig ability (log odds, SE) to change BW class between birth and finisher (A) and weaning and finisher (B). (DOCX 95 kb) [file 40813_2018_98_MOESM6_ESM.docx]

**

**

**

**

**

**

**

**

**

**

**

**

**

**

**

**

**

**

**

*■ µ 145*

*± 29.1*

*■ µ 188*

*± 19.2*

*■ µ 220*

*± 22.5*

*■ µ 269*

*± 31.9*

*■ µ 178*

*± 44.8*

*■ µ 204*

*± 48.4*

*■ µ 216*

*± 49.3*

*■ µ 220*

*± 56.8*

4

3

2

1

4

3

2

1

**Figure S3**

Effect of pre-weaning ADG (d 0 to 28) on pig ability (log odds, SE) to change BW class between birth and finisher (**A**) and weaning and finisher (**B**). Within batch, BW classes were created using percentiles (25%) resulting in 4 groups. Class 1 represents the lightest pig, class 4 the heaviest. The different colours represent BW class at birth (A) or weaning (B), with respectively class 1 **■**, class 2 **■**, class 3 **■**, and class 4 **■.** Coefficients were estimated for each BW class separate. Pigs were weighed at birth (d 0), at weaning (d 27.7; SD = 1.07), and at finisher (d 98.8; SD = 0.937). The µ ± SED on the x-axis represent the average of the characteristic of interest for each BiW or WW class. ** (*P* < 0.05), * (*P* < 0.10)
