# Supplementary material for: Once small always small? To what extent morphometric characteristics and post-weaning starter regime affect pig lifetime growth performance
Source: Porcine Health Manag. 2018 Jul 23;4:21. doi: 10.1186/s40813-018-0098-1 (PMC6055348; doi:10.1186/s40813-018-0098-1)
Supplement: Supplementary file 7 — Table S4. Statistical significance (P - value) of the different predictor variables fitted in the univariate models for piglets of a different birth weight (BiW) class for ADG (g/d) between d 0 to 28 and d 28 to 99. (DOCX 36 kb) [file 40813_2018_98_MOESM7_ESM.docx]

**Table S4**

Statistical significance (*P* - value) of the different predictor variables fitted in the univariate models for piglets of a different birth weight (BiW) class for ADG (g/d) between d 0 to 28 and d 28 to 99. Within batch, BiW classes were created retrospectively using percentiles resulting in 4 (25%) classes. Class 1 represents the lightest pig, class 4 the heaviest. Morphometric measurements were taken within 12 h post-partum, pigs were weighed at birth (d 0), at weaning (d 27.7; SD = 1.07), and at finisher (d 98.8; SD = 0.937).

|  | d 0 - 28 | | | |  | d 28 - 99 | | | |
| --- | --- | --- | --- | --- | --- | --- | --- | --- | --- |
| Predictor variable | Class 1 | Class 2 | Class 3 | Class 4 |  | Class 1 | Class 2 | Class 3 | Class 4 |
| Birth weight, kg | **<0.001** | **0.017** | 0.615 | **<0.001** |  | **<0.001** | 0.423 | 0.221 | 0.256 |
| Relative birth weight^1^ | **<0.001** | 0.886 | 0.913 | **<0.001** |  | **<0.001** | 0.555 | 0.355 | 0.449 |
| Weaning weight, kg | - | - | - | - |  | **<0.001** | **0.005** | **0.004** | **<0.001** |
| Pre-weaning ADG, g/day | - | - | - | - |  | **<0.001** | **0.014** | **0.011** | **<0.001** |
| Gender | 0.941 | **0.019** | 0.702 | 0.449 |  | 0.346 | 0.870 | 0.846 | 0.698 |
| Starter regime | - | - | - | - |  | 0.186 | 0.296 | 0.568 | 0.891 |
| Crown to rump length, cm | 0.631 | 0.703 | 0.940 | **0.002** |  | **0.012** | 0.653 | 0.729 | 0.664 |
| Snout to ear length, cm | 0.702 | **0.050** | 0.699 | **0.003** |  | 0.104 | 0.944 | 0.791 | 0.051 |
| Abdominal circumference, cm | 0.201 | **0.040** | **0.046** | **0.009** |  | 0.050 | 0.601 | 0.795 | 0.426 |
| Cranial circumference, cm | **0.007** | 0.815 | 0.462 | 0.085 |  | 0.073 | 0.745 | 0.583 | 0.437 |
| Body mass index^2^, kg/m^2^ | **<0.001** | 0.370 | 0.724 | 0.861 |  | **0.040** | 0.543 | 0.191 | 0.194 |
| Ponderal index^3^, kg/m^3^ | **0.005** | 0.527 | 0.810 | 0.237 |  | 0.486 | 0.658 | 0.268 | 0.246 |
| Birth weight: Cranial circumference, kg/cm | **<0.001** | **0.018** | 0.990 | **<0.001** |  | **<0.001** | 0.362 | 0.379 | 0.366 |
| Snout to ear length: Birth weight, cm/kg | **<0.001** | 0.929 | 0.974 | **<0.001** |  | **<0.001** | 0.656 | 0.272 | 0.679 |
| Litter size pre-weaning^4^ | 0.120 | 0.064 | 0.166 | 0.632 |  | 0.825 | 0.174 | 0.159 | **0.012** |
| Group size post-weaning^4^ | - | - | - | - |  | 0.827 | 0.797 | 0.778 | **0.023** |

^1^ Relative birth weight = (Birth weight piglet/ mean birth weight birth litter)

^2^ Body mass index = birth weight (kg)/[crown rump length (m)]^2^

^3^ Ponderal index = birth weight (kg)/[crown rump length (m)]^3^

^4^ Pre-weaning litter size/ group size post-weaning = [(total time (h) piglets reside within litter/ pen)/24 h]/ total period in d
