# Supplementary material for: Once small always small? To what extent morphometric characteristics and post-weaning starter regime affect pig lifetime growth performance
Source: Porcine Health Manag. 2018 Jul 23;4:21. doi: 10.1186/s40813-018-0098-1 (PMC6055348; doi:10.1186/s40813-018-0098-1)
Supplement: Supplementary file 8 — Figure S4. Effect of various morphometric characteristics on pig ability (log odds ± SE) to change BW class between weaning (WW) and finisher (FW). (DOCX 232 kb) [file 40813_2018_98_MOESM8_ESM.docx]

**

**

*

**

**

**

**

*■ µ 24.1*

*± 2.18*

*■ µ 25.1*

*± 2.05*

*■ µ 25.5*

*± 1.81*

*■ µ 26.4*

*± 1.84*

*■ µ 20.9*

*± 2.35*

*■ µ 22.0*

*± 2.03*

*■ µ 22.5*

*± 1.95*

*■ µ 23.5*

*± 1.78*

*

*

**

**

**

**

**

**

**

**

**

*

*

**

**

**

*

*

**

**

**

**

**

**

*■ µ 9.76*

*± 0.652*

*■ µ 10.1*

*± 0.620*

*■ µ 10.0 ± 0.599*

*■ µ 10.3*

*± 0.639*

*■ µ 20.6*

*± 1.50*

*■ µ 21.2*

*± 1.23*

*■ µ 21.4*

*± 1.15*

*■ µ 22.0*

*± 1.01*

**

**

**

**

**

**

**

**

- **

**

**

**

*

**

**

**

*■ µ 89.7*

*± 14.4*

*■ µ 92.4*

*± 15.0*

*■ µ 93.3*

*± 15.8*

*■ µ 93.0*

*± 15.1*

*■ µ 21.6*

*± 3.39*

*■ µ 23.0*

*± 3.11*

*■ µ 23.6*

*± 3.19*

*■ µ 24.4*

*± 3.07*

**

**

**

**

*

**

**

**

**

**

*

**

**

**

**

**

**

**

**

**

*■ µ 0.0613*

*± 0.0125*

*■ µ 0.0684*

*± 0.0108*

*■ µ 0.0710*

*± 0.00912*

*■ µ 0.0768*

*± 0.00949*

*■ µ 8.15*

*± 2.07*

*■ µ 7.14*

*± 1.34*

*■ µ 6.72*

*± 1.05*

*■ µ 6.18*

*± 0.870*

1

1

4

3

2

2

4

3

**Figure S4**

Effect of various morphometric characteristics on pig ability (log odds ± SE) to change BW class between weaning (WW) and finisher (FW). Within batch, BW classes were created using percentiles (25%) resulting in 4 groups: class 1 represents the lightest pig, class 4 the heaviest. The different colours represent WW class 1 **■**, class 2 **■**, class 3 **■**, and class 4 **■.** Coefficients were estimated for each WW class separate. Morphometric measurements were taken within 12 h post-partum and pigs WW and FW were taken at respectively d 27.7 (SD = 1.07) and d 98.8 (SD = 0.937). The µ ± SED on the x-axis represent the average of the characteristic of interest for each WW class. ** (*P* < 0.05), * (*P* < 0.10)
