# Supplementary material for: Once small always small? To what extent morphometric characteristics and post-weaning starter regime affect pig lifetime growth performance
Source: Porcine Health Manag. 2018 Jul 23;4:21. doi: 10.1186/s40813-018-0098-1 (PMC6055348; doi:10.1186/s40813-018-0098-1)
Supplement: Supplementary file 9 — Table S5. Rank correlations between predictor variables for piglets of a different weaning weight (WW) class. (DOCX 42.8 kb) [file 40813_2018_98_MOESM9_ESM.docx]

**Table S5**

Rank correlations between predictor variables for piglets of a different weaning weight (WW) class. Within batch, WW classes were created retrospectively using percentiles resulting in 4 (25%) classes. Class 1 represents the lightest pig, class 4 the heaviest. Numbers in bold were variables that were considered highly correlated (*r* > +/- 0.70).^1,2^

**WW class 1**

| Predictor variable | BiW | Rel BiW | WW | ADG | CRL | HL | AC | CC | BMI | PI | BiW: CC | HL: BiW |
| --- | --- | --- | --- | --- | --- | --- | --- | --- | --- | --- | --- | --- |
| Birth weight (BiW), kg | - |  |  |  |  |  |  |  |  |  |  |  |
| Relative Birth weight (Rel BiW)^3^ | **0.854** | - |  |  |  |  |  |  |  |  |  |  |
| Weaning weight (WW), kg | 0.240 | 0.215 | - |  |  |  |  |  |  |  |  |  |
| Pre-weaning ADG (ADG), g/day | -0.151 | *ns* | **0.906** | - |  |  |  |  |  |  |  |  |
| Crown to rump length (CRL), cm | **0.825** | **0.750** | 0.183 | -0.124 | - |  |  |  |  |  |  |  |
| Snout to ear length (HL), cm | 0.571 | 0.548 | 0.109 | *ns* | 0.493 | - |  |  |  |  |  |  |
| Abdominal circumference (AC), cm | **0.817** | **0.748** | 0.140 | -0.180 | 0.692 | 0.444 | - |  |  |  |  |  |
| Cranial circumference (CC), cm | **0.870** | **0.733** | 0.225 | -0.119 | **0.725** | 0.498 | **0.780** | - |  |  |  |  |
| Body mass index^4^(BMI), kg/m^2^ | **0.757** | 0.620 | 0.227 | *ns* | 0.271 | 0.425 | 0.624 | 0.669 | - |  |  |  |
| Ponderal index^5^ (PI), kg/m^3^ | 0.282 | 0.195 | 0.131 | *ns* | -0.290 | 0.437 | 0.231 | 0.249 | **0.836** | - |  |  |
| BiW: CC, kg/cm | **0.983** | **0.856** | 0.241 | -0.140 | **0.819** | 0.571 | **0.789** | **0.775** | **0.742** | 0.273 | - |  |
| HL: BiW, cm/kg | **-0.932** | **-0.828** | -0.272 | *ns* | **-0.797** | -0.399 | **-0.807** | **-0.847** | **-0.723** | -0.273 | **-0.925** | - |

**WW class 2**

| Predictor variable | BiW | Rel BiW | WW | ADG | CRL | HL | AC | CC | BMI | PI | BiW: CC | HL: BiW |
| --- | --- | --- | --- | --- | --- | --- | --- | --- | --- | --- | --- | --- |
| Birth weight (BiW), kg | - |  |  |  |  |  |  |  |  |  |  |  |
| Relative Birth weight (Rel BiW)^3^ | **0.730** | - |  |  |  |  |  |  |  |  |  |  |
| Weaning weight (WW), kg | 0.127 | *ns* | - |  |  |  |  |  |  |  |  |  |
| Pre-weaning ADG (ADG), g/day | -0.390 | -0.264 | **0.758** | - |  |  |  |  |  |  |  |  |
| Crown to rump length (CRL), cm | **0.746** | 0.604 | *ns* | -0.267 | - |  |  |  |  |  |  |  |
| Snout to ear length (HL), cm | 0.526 | 0.413 | 0.113 | -0.115 | 0.512 | - |  |  |  |  |  |  |
| Abdominal circumference (AC), cm | 0.692 | 0.549 | *ns* | -0.286 | 0.281 | 0.426 | - |  |  |  |  |  |
| Cranial circumference (CC), cm | **0.796** | 0.534 | 0.189 | -0.272 | 0.578 | 0.422 | 0.603 | - |  |  |  |  |
| Body mass index^4^(BMI), kg/m^2^ | 0.604 | 0.405 | *ns* | -0.248 | *ns* | 0.185 | 0.354 | 0.503 | - |  |  |  |
| Ponderal index^5^ (PI), kg/m^3^ | 0.134 | *ns* | *ns* | *ns* | -0.543 | *ns* | *ns* | 0.132 | **0.869** | - |  |  |
| BiW: CC, kg/cm | **0.974** | **0.742** | *ns* | -0.392 | **0.739** | 0.516 | 0.664 | 0.644 | 0.581 | 0.121 | - |  |
| HL: BiW, cm/kg | **-0.930** | **-0.724** | *ns* | 0.387 | -0.682 | -0.281 | -0.655 | **-0.749** | -0.608 | -0.173 | **-0.918** | - |

**WW class 3**

| Predictor variable | BiW | Rel BiW | WW | ADG | CRL | HL | AC | CC | BMI | PI | BiW: CC | HL: BiW |
| --- | --- | --- | --- | --- | --- | --- | --- | --- | --- | --- | --- | --- |
| Birth weight (BiW), kg | - |  |  |  |  |  |  |  |  |  |  |  |
| Relative Birth weight (Rel BiW)^3^ | 0.670 | - |  |  |  |  |  |  |  |  |  |  |
| Weaning weight (WW), kg | 0.123 | *ns* | - |  |  |  |  |  |  |  |  |  |
| Pre-weaning ADG (ADG), g/day | -0.301 | -0.215 | **0.805** | - |  |  |  |  |  |  |  |  |
| Crown to rump length (CRL), cm | 0.656 | 0.478 | *ns* | -0.246 | - |  |  |  |  |  |  |  |
| Snout to ear length (HL), cm | 0.497 | 0.386 | *ns* | -0.183 | 0.420 | - |  |  |  |  |  |  |
| Abdominal circumference (AC), cm | 0.675 | 0.513 | *ns* | -0.217 | 0.487 | 0.306 | - |  |  |  |  |  |
| Cranial circumference (CC), cm | **0.828** | 0.522 | 0.189 | 0.175 | 0.531 | 0.421 | 0.593 | - |  |  |  |  |
| Body mass index^4^(BMI), kg/m^2^ | 0.576 | 0.373 | 0.128 | -0.111 | -0.229 | 0.196 | 0.354 | 0.494 | - |  |  |  |
| Ponderal index^5^ (PI), kg/m^3^ | 0.192 | *ns* | *ns* | *ns* | -0.613 | *ns* | *ns* | 0.176 | **0.909** | - |  |  |
| BiW: CC, kg/cm | **0.972** | 0.674 | *ns* | -0.321 | 0.638 | 0.472 | 0.636 | 0.669 | 0.559 | 0.176 | - |  |
| HL: BiW, cm/kg | **-0.919** | -0.644 | -0.148 | 0.247 | -0.593 | -0.187 | -0.655 | **-0.780** | -0.561 | -0.209 | **-0.898** | - |

**WW class 4**

| Predictor variable | BiW | Rel BiW | WW | ADG | CRL | HL | AC | CC | BMI | PI | BiW: CC | HL: BiW |
| --- | --- | --- | --- | --- | --- | --- | --- | --- | --- | --- | --- | --- |
| Birth weight (BiW), kg | - |  |  |  |  |  |  |  |  |  |  |  |
| Relative Birth weight (Rel BiW)^3^ | 0.598 | - |  |  |  |  |  |  |  |  |  |  |
| Weaning weight (WW), kg | 0.282 | 0.187 | - |  |  |  |  |  |  |  |  |  |
| Pre-weaning ADG (ADG), g/day | *ns* | *ns* | **0.908** | - |  |  |  |  |  |  |  |  |
| Crown to rump length (CRL), cm | 0.630 | 0.363 | 0.170 | **0.968** | - |  |  |  |  |  |  |  |
| Snout to ear length (HL), cm | 0.432 | 0.203 | 0.242 | 0.145 | 0.315 | - |  |  |  |  |  |  |
| Abdominal circumference (AC), cm | 0.627 | 0.489 | 0.191 | *ns* | 0.487 | 0.256 | - |  |  |  |  |  |
| Cranial circumference (CC), cm | **0.747** | 0.447 | 0.294 | *ns* | 0.470 | 0.376 | 0.511 | - |  |  |  |  |
| Body mass index^4^(BMI), kg/m^2^ | 0.525 | 0.337 | 0.146 | *ns* | -0.322 | 0.184 | 0.233 | 0.397 | - |  |  |  |
| Ponderal index^5^ (PI), kg/m^3^ | 0.133 | *ns* | *ns* | *ns* | 0.675 | *ns* | *ns* | *ns* | **0.912** | - |  |  |
| BiW: CC, kg/cm | **0.966** | 0.582 | 0.237 | *ns* | 0.617 | 0.401 | 0.591 | 0.553 | 0.501 | 0.121 | - |  |
| HL: BiW, cm/kg | **-0.900** | -0.592 | -0.178 | *ns* | -0.568 | *ns* | -0.591 | -0.656 | -0.483 | -0.127 | **-0.881** | - |

^1^ Pearson correlation test was used to estimate correlations between continuous variables that were normally distributed. Variables with a high correlation (*r* ≥ 0.70) are in bold. Morphometric measurements were taken within 12 h post-partum, pigs were weighed at birth (d 0) and at weaning (d 27.7; SD = 1.07).

^2^ *ns* = not significant (*P* > 0.05)

^3^ Relative birth weight = (Birth weight piglet/ mean birth weight birth litter)

^4^ Body mass index = birth weight (kg)/[crown rump length (m)]^2^

^5^ Ponderal index = birth weight (kg)/[crown rump length (m)]^3^
