# Supplementary material for: Once small always small? To what extent morphometric characteristics and post-weaning starter regime affect pig lifetime growth performance
Source: Porcine Health Manag. 2018 Jul 23;4:21. doi: 10.1186/s40813-018-0098-1 (PMC6055348; doi:10.1186/s40813-018-0098-1)
Supplement: Supplementary file 10 — Table S6. Statistical significance (P - value) of the different predictor variables fitted in the univariate models for piglets of a different weaning weight (WW) class for ADG (g/d) between d 28 and 99. (DOCX 35 kb) [file 40813_2018_98_MOESM10_ESM.docx]

**Table S6**

Statistical significance (*P* - value) of the different predictor variables fitted in the univariate models for piglets of a different weaning weight (WW) class for ADG (g/d) between d 28 and 99. Within batch, WW classes were created retrospectively using percentiles resulting in 4 (25%) classes. Class 1 represents the lightest pig, class 4 the heaviest. Morphometric measurements were taken within 12 h post-partum, pigs were weighed at birth (d 0), at weaning (d 27.7; SD = 1.07), and at finisher (d 98.8; SD = 0.937).

|  | d 28 - 99 | | | |
| --- | --- | --- | --- | --- |
| Predictor variable | Class 1 | Class 2 | Class 3 | Class 4 |
| Birth weight, kg | **<0.001** | **<0.001** | 0.074 | **0.018** |
| Relative birth weight^1^ | **<0.001** | **0.002** | **0.028** | 0.056 |
| Weaning weight, kg | **<0.001** | 0.610 | 0.567 | 0.742 |
| Pre-weaning ADG, g/day | **<0.001** | **<0.001** | 0.445 | 0.553 |
| Gender | 0.086 | **0.025** | 0.942 | 0.159 |
| Starter regime | 0.243 | 0.890 | 0.835 | 0.202 |
| Crown to rump length, cm | **0.021** | **0.005** | 0.132 | 0.584 |
| Snout to ear length, cm | 0.084 | **0.012** | 0.434 | **0.014** |
| Abdominal circumference, cm | **<0.001** | **0.001** | 0.363 | **0.041** |
| Cranial circumference, cm | **<0.001** | **0.006** | 0.109 | 0.503 |
| Body mass index^2^, kg/m^2^ | **<0.001** | **0.040** | 0.427 | **0.032** |
| Ponderal index^3^, kg/m^3^ | **0.001** | 0.772 | 0.984 | 0.146 |
| Birth weight: Cranial circumference, kg/cm | **<0.001** | **<0.001** | 0.077 | **0.009** |
| Snout to ear length: Birth weight, cm/kg | **<0.001** | **0.002** | 0.057 | 0.201 |
| Litter size pre-weaning^4^ | 0.776 | **0.041** | 0.445 | 0.055 |
| Group size post-weaning^4^ | 0.863 | 0.784 | 0.389 | 0.545 |

^1^ Relative birth weight = (Birth weight piglet/ mean birth weight birth litter)

^2^ Body mass index = birth weight (kg)/[crown rump length (m)]^2^

^3^ Ponderal index = birth weight (kg)/[crown rump length (m)]^3^

^4^ Pre-weaning litter size/ group size post-weaning = [(total time (h) piglets reside within litter/ pen)/24 h]/ total period in d
